# High-throughput long paired-end sequencing of a Fosmid library by PacBio

**DOI:** 10.1186/s13007-019-0525-6

**Published:** 2019-11-26

**Authors:** Zhaozhao Dai, Tong Li, Jiadong Li, Zhifei Han, Yonglong Pan, Sha Tang, Xianmin Diao, Meizhong Luo

**Affiliations:** 1 College of Life Science and Technology, Huazhong Agricultural University, Wuhan, 430070 China; 2 Institute of Crop Sciences, Chinese Academy of Agricultural Sciences, Beijing, 10081 China

**Keywords:** Fosmid, Long paired-end, Mate-pair, PacBio, *Ampicillin* resistance gene tag, De novo assembly, Structural rearrangement, Assembly error

## Abstract

**Background:**

Large insert paired-end sequencing technologies are important tools for assembling genomes, delineating associated breakpoints and detecting structural rearrangements. To facilitate the comprehensive detection of inter- and intra-chromosomal structural rearrangements or variants (SVs) and complex genome assembly with long repeats and segmental duplications, we developed a new method based on single-molecule real-time synthesis sequencing technology for generating long paired-end sequences of large insert DNA libraries.

**Results:**

A Fosmid vector, pHZAUFOS3, was developed with the following new features: (1) two 18-bp non-palindromic I*-*SceI sites flank the cloning site, and another two sites are present in the skeleton of the vector, allowing long DNA inserts (and the long paired-ends in this paper) to be recovered as single fragments and the vector (~ 8 kb) to be fragmented into 2–3 kb fragments by I-SceI digestion and therefore was effectively removed from the long paired-ends (5–10 kb); (2) the *chloramphenicol* (Cm) resistance gene and replicon (*ori*V), necessary for colony growth, are located near the two sides of the cloning site, helping to increase the proportion of the paired-end fragments to single-end fragments in the paired-end libraries. Paired-end libraries were constructed by ligating the size-selected, mechanically sheared pooled Fosmid DNA fragments to the *Ampicillin* (Amp) resistance gene fragment and screening the colonies with Cm and Amp. We tested this method on yeast and *Setaria italica* Yugu1. Fosmid-size paired-ends with an average length longer than 2 kb for each end were generated. The N50 scaffold lengths of the de novo assemblies of the yeast and *S. italica* Yugu1 genomes were significantly improved. Five large and five small structural rearrangements or assembly errors spanning tens of bp to tens of kb were identified in *S. italica* Yugu1 including deletions, inversions, duplications and translocations.

**Conclusions:**

We developed a new method for long paired-end sequencing of large insert libraries, which can efficiently improve the quality of de novo genome assembly and identify large and small structural rearrangements or assembly errors.

## Background

The development of DNA sequencing technology has a short and rich history, and there have been many advancements in just over 40 years [1]. With Sanger's electrophoresis (the first generation) sequencing technology [2], the door to DNA sequencing was opened with its long read length and high precision, but its high cost and low throughput limits its development [3, 4]. Massively parallel genome-sequencing technologies [4], with their low cost, high throughput, high accuracy and other characteristics, have become the mainstay of biological sequencing, except that short read lengths seriously hinder the study of large and complex genomes containing long repeats [5]. Single-molecule real-time synthesis and sequencing technology such as PacBio [6, 7] and Oxford Nanopore Technologies [8–10] are new leading technologies with high throughput, long read length and other advantages, that create a new era of biological sequencing, although their disadvantages, such as a high error rate, can not be ignored. Currently, these DNA sequencing technologies are being rapidly developed and updated, and are widely used in de novo assembly [3, 4], individual genome resequencing [11–14], clinical applications such as non-invasive prenatal testing [15, 16], and counting devices for a wide range of biochemical or analytical phenomena [1].

Genomic libraries are collections of genomic DNA from a certain species that has been fragmented into specific sizes by biological, chemical or physical disruption. They are important tools and materials for molecular cloning, genomic structure and functional characteristic research [17]. Among genomic libraries, large-insert genomic libraries, such as Fosmid libraries (average insert approximately 40 kb) [18] and BAC library (average insert > 100 kb) [19–21], are widely used in physical map construction, genome-wide sequencing, comparative genomics research, and genomic resource conservation due to their capacity for long lengths of foreign DNA fragments.

Paired-end (or mate-pair) sequencing technology using genomic libraries with different inserts to obtain paired-end sequences through different sequencing technologies- plays an important role in the field of biological sequencing. For example, the BAC library clones’ end sequences are generated through Sanger sequencing technology to construct physical maps that help resolve long repeats and segmental duplications and provide long-range connectivity in shotgun assemblies of complex genomes [22–24]. Fosmids are shorter than BACs but much easier to generate. Therefore, mate-pair Fosmid library clones’ end sequences [25, 26] based on the Illumina sequencing platform enable the detection of structural variation predominantly mediated by repetitive elements such as insertions, deletions, and inversions [4, 27–29], which are commonly larger than 1 kb and are difficult to identify using conventional small insert paired-end libraries (300–500 bp) [30–32]. This method also enables the identification of unique sequences in the flanking regions of repetitive elements that potentially reveal precise structural variants breakpoint(s). In addition, data generated by paired-end libraries facilitates clinical application and shows that when the physical coverage increases, the required minimum read depth decreases [26, 32]. Moreover, paired-end sequences of Fosmid and BAC libraries have made significant contributions in identifying long range structural variations in inter- or intra- chromosomes and in assessing the quality of whole genome assemblies, even correcting misassemblies and reducing contig numbers [33–35].

However, the first and second generation sequencing platforms can not generate DNA sequences longer than 1 kb, and the cost of the first generation sequencing platform is very high. Thus, the short read pairs (< 1 kb) generated by these paired-end sequencing technologies are limited in the assembly of complex genomes, and repetitive regions (> 1 kb) are usually missing or misassembled, leading to fragmented and incomplete genomes. Therefore, longer paired-end reads are required.

Recently, new technologies that can also be used for genome assembly such as 10× Genomics, Hi-C and BioNano are being developed. They have their own characteristics and applications. Data from 10× Genomics are widely used in de novo whole genome assembly [36, 37], assisting genome assembly [38] and detecting structural variants [39, 40] because of large spans (> 50 kb) and a low cost. Hi-C related articles such as identifying target genes [41], revealing structural remodeling [42] and analyzing enhancer expression [43], have risen exponentially since 2017. The Hi-C technology also has been widely used in assisting genome assembly [44, 45]. BioNano improves genome assembly [46] and detects genome-wide SVs [47] based on single-molecule optical mapping technologies with its long connective data. Single molecule sequencing technologies have become routine in genomics. However, the paired-end sequencing of fosmid and BAC clones, 10× genomics, Hi-C, and Bionano optical mapping provide long connective data that are necessary for genome assembly and regularly used across the plant tree of life.

Although many methods have been developed as described above and applied in the study of genomic sequencing, the biological genome is difficult to explore clearly with just one or a few methods, especially for large animal and plant genomes with a high GC content and long repeat sequences. Therefore, the combination of different methods and mutual verification has become the mainstay of current genome sequencing. Hence, we developed a new method for genome sequencing to break the limitation that traditional jumping libraries can not generate reads with an average length longer than 1 kb. Our method provides an alternative way to assist genome assembly and has an advantage that the interested large fragment clones can be screened out by their corresponding end sequences. The utilities of the method in de novo assembly and structural rearrangement detection were tested on the yeast and *S. italica* Yugu1 genomes.

## Results

### The pipeline of high-throughput long paired-end sequencing of a Fosmid library

**Fig. 1:**
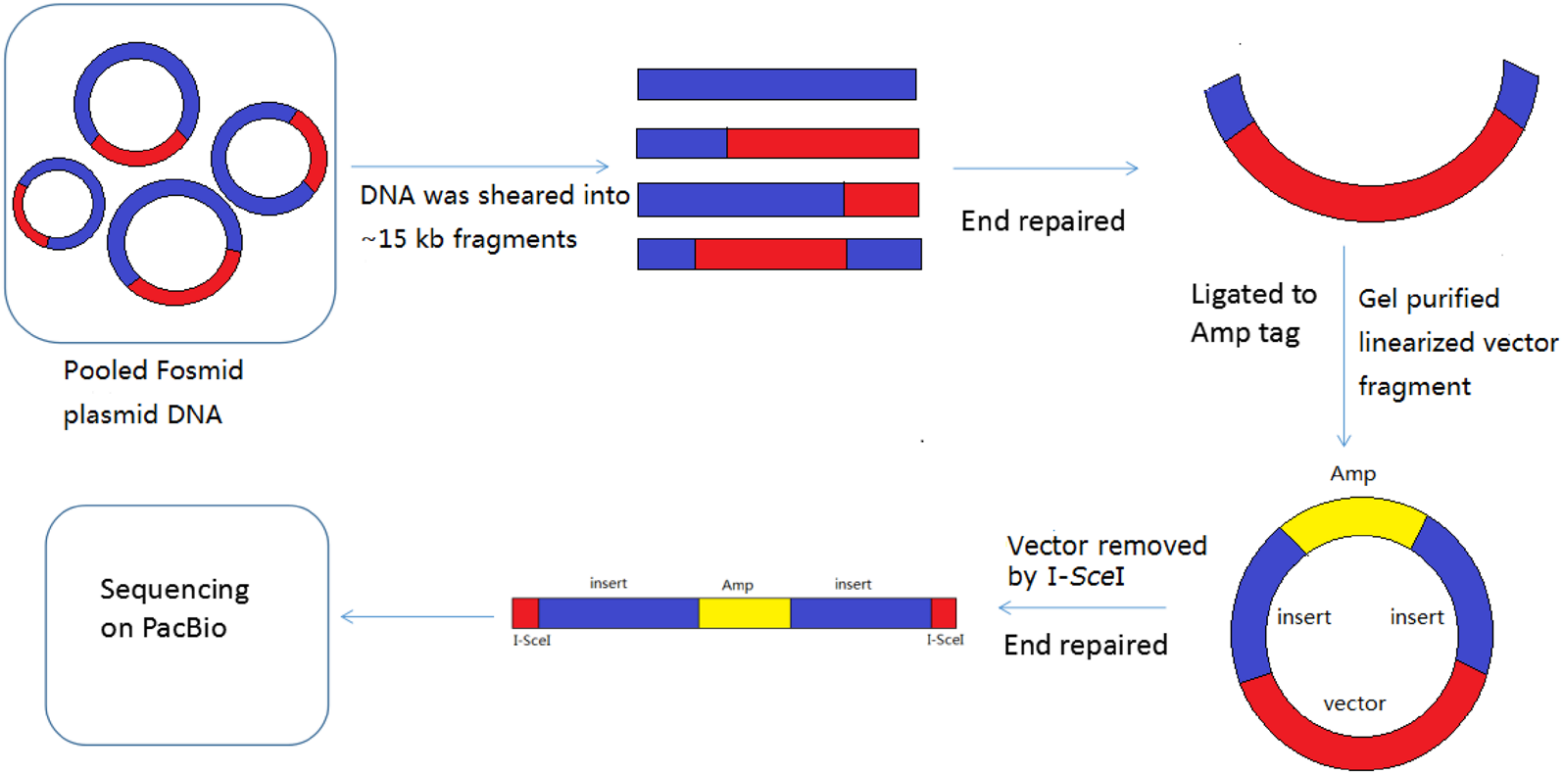
The pipeline of Fosmid-size long paired-end library construction. The red area represents the vector, the blue area represents the large inserted genomic fragment, and the yellow area represents the *Ampicillin* resistance gene tag. The Fosmid clones were pooled together, and DNA was extracted for paired-end library construction. Pooled Fosmid plasmid DNA was sheared into ~ 15 kb fragments by g-TUBE (Covaris). It generated insert only, vector with single-ends and vector with paired-ends. Then, these DNA fragments were end repaired and gel purified for ligation with the *Ampicillin* resistance gene tag. Although all fragments could be ligated to the *Ampicillin* resistance gene tag, only those containing the chloramphenicol resistant gene and *ori*V ligated to an Amp tag were screened out with double resistance to chloramphenicol and ampicillin after transformation. Finally, the vector was removed by I-SceI and the paired-end fragments with the Amp tag were sequenced on PacBio

To enrich the approaches of genome sequencing, we developed a new method to generate high-throughput long paired-end fragments of a Fosmid library. Figure 1 shows the pipeline of the method. A Fosmid library was constructed. Pooled Fosmid DNA was sheared into 13–18 kb fragments and separated by pulse field gel electrophoresis (PFGE). Size selected DNA fragments were recovered by electroelution, end-repaired and ligated to the *Ampicillin* resistance gene label. Colonies transformed with the paired-end fragments containing the vector and the Amp tag were screened by chloramphenicol and ampicillin. Then, the vector was removed by I*-*SceI, and the paired-end fragments containing Amp were recovered and sequenced on the PacBio Sequel platform.

### The first modification of the Fosmid vector based on pcc2FOS

**Fig. 2:**
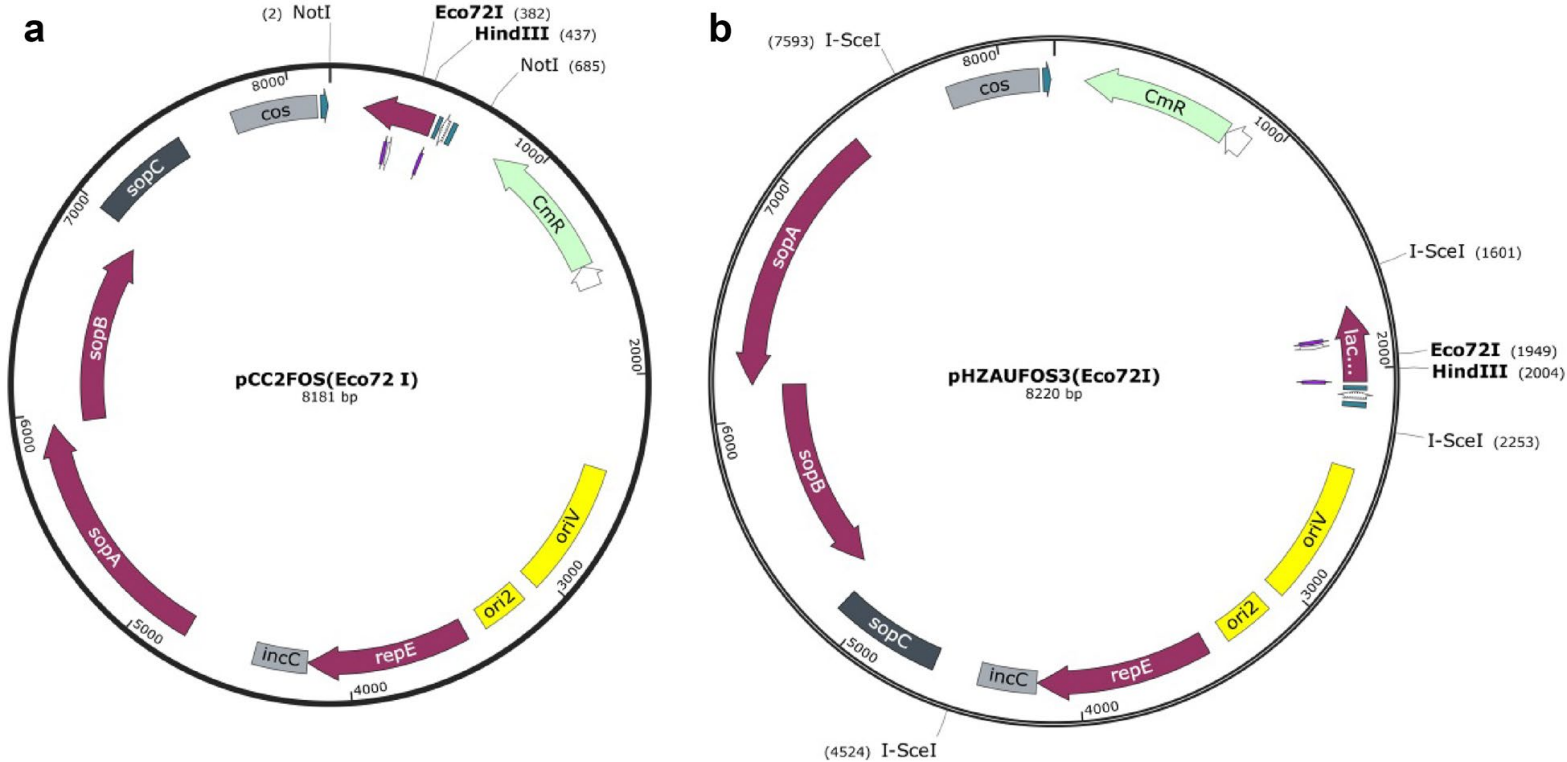
The maps of the vectors pcc2FOS and pHZAUFOS3.A is the map of pcc2FOS. *Not*I was used to release the insert and the *lac*Z fragment was outside of CmR and *ori*V. B is the map of pHZAUFOS3. The *Lac*Z fragment was moved between CmR and *ori*V; the two I*-*SceI sites adjacent to *Lac*Z were used to release the insert, and another two I*-*SceI sites were used to break the vector skeleton into small fragments (2–3 kb)

In our new method, the recovery of complete long paired ends as single fragments from the paired-end library was critical. Therefore, we replaced the two 8-bp *Not*I restriction sites flanking the *Lac*Z fragment harboring the cloning sites in pcc2FOS (Fig. 2a) with the 18-bp homing endonuclease I-SceI sites by PCR using the primers P1 (5′-attaccctgttatccctaGTCGGGGCTGGCTTAACTAT- 3′) and P2 (5′-attaccctgttatccctaTTCGCGTTGGCCGATTCATT-3′) containing the I-SceI sites at the 5′ ends, resulting in the fragment named A (Additional file 1: Figure S1).

In the pipeline, mechanical interruption was adopted to break the pooled Fosmid DNA. This resulted in 3 main types of fragments: (1) Fragments containing the entire vector sequence and the paired-end insert sequence (2) fragments containing part of or the entire vector sequence and single-end insert sequence, and (3) fragments containing only the insert sequence without the vector sequence. Only the fragments containing both the replicon (*ori*V) and *Chloramphenicol* resistant gene (CmR) in vector as in (1) and (2) could be screened out by transformation (Additional file 1: Figure S2). However, *ori*V and CmR were both on the same side of the multiple cloning sites in pcc2FOS, which resulted in a high proportion of single ends in our prediction. To improve efficiency and reduce the cost of sequencing, the proportion of (1) must be increased. Thus, we moved the *Lac*Z fragment containing multiple cloning sites to the position between the *ori*V and CmR. The pcc2FOS vector was digested by *Not*I, and the pcc2FOS backbone without *Lac*Z was recovered, self-ligated and propagated in *E. coli* EPI300.-T1R. Then, new PCR primers, P3 (5′-ATTCAAATCGTTTTCGTTACCGC-3′) and P4 (5′-ATGCCTTCAGGAACAATAGAAATCT-3′), with sequences complementary to the area between *ori*V and CmR were used to generate the skeleton of the vector pcc2FOS, named B (Additional file 1: Figure S1). The PCR products A and B were ligated, resulting in pHZAUFOS2 (Additional file 1: Figure S3).

### Preliminary test of the method for Fosmid long paired-end sequencing

To test the new Fosmid paired-end sequencing strategy, we used pHZAUFOS2 to construct two Fosmid libraries: Y1 for *Saccharomyces cerevisiae* S288C and S1 for *Setaria italica* Yugu1. The library sizes were estimated to be 1.2 million colony-forming units (cfu) and 90 thousand colony-forming units (cfu), corresponding to 15× physical genome coverage and 10× physical genome coverage for Y1 and S1, respectively. Fosmid clones of each library were amplified in bulk by overnight liquid culture at 37 °C, and pooled Fosmid DNA was prepared. A paired-end library was constructed with pooled Fosmid DNA. Again, pooled paired-end library DNA was extracted, digested with I-SceI and size-selected on PFGE gels. Paired ends were recovered and sequenced on Frasergene's PacBio RSII platform. The reads were aligned to the reference genomes of the *S. cerevisiae* S288C and *S. italica* Yugu1 (Additional file 2: Table S1).

We obtained a total of 35,510 clean end subreads from library Y1 after removing reads shorter than 50 bp. The N50 of each end was almost 3 kb, and the longest subread was 15 kb (Table 1, library Y1). These clean end reads were used for alignment with the reference genome *S. cerevisiae* S288C. After removing those unaligned reads, single-end aligned reads, chimaeras and reads aligned to multiple places, 25,812 reads (73%) were obtained as unambiguously placed paired ends. A total of 22,192 (86%) of 25,812 reads were unambiguously mapped in the expected spacing (20–50 kb) and correct orientation (convergent) on the reference genome. On average, these correct Fosmid jumps were 38 kb in length with a standard deviation of 2.2 kb. After deduplication, we recovered a total of 3067 unique Fosmid-size jumps, covering approximately tenfold of the *S. cerevisiae* S288C genome.

**Table 1:** Summarized statistics for the four Fosmid-size paired-end libraries

Sample		FES^a^ number	FES-1^b^ N50 (bp)	FES-1 average length (bp)	FES-1 total bases (bp)	FES-2^c^ N50 (bp)	FES-2 average length (bp)	FES-2 total bases (bp)
Y1	S288C_1	35,510	3066	2004	71,170,214	3112	2014	71,513,294
Y2	S288C_2	17,844	2742	1884	33,626,713	2709	1845	32,925,281
	Yugu1_1	20,119	2466	1656	33,311,652	2435	1642	33,039,852
	Yugu1_2	5476	2316	1663	9,104,650	2327	1618	8,862,453
S1	Yugu1_3	295	2381	1725	508,797	2509	1889	557,384
	Yugu1_4	21,657	3484	2220	48,077,465	3345	2180	47,212,605
	Yugu1_5	4546	3391	2419	10,995,363	3455	2449	11,133,474
	Yugu1_6	15,127	2556	1642	24,838,345	2496	1613	24,405,221
S2	Yugu1_t	75,047	2853	2060	154,610,364	2850	2057	154,381,829

^a^*FES* Fosmid end sequence, ^b^*FES-1* Fosmid left-end sequence, ^c^*FES-2* Fosmid right-end sequence

We also obtained a total of 67,220 clean subreads from library S1. The N50 of each end was 2.8 kb (Table 1, library S1). These clean end reads were used for alignment with the reference genome *S. italica* Yugu1. After removing those unaligned reads, single-end aligned reads, chimaeras and reads aligned to multiple places, 41,998 (63%) reads were obtained as unambiguously placed paired ends. A total of 36,969 (88%) of 41,998 reads had correct Fosmid jumps (20–50 kb). After deduplication, we recovered a total of 13,334 unique Fosmid-sized jumps, covering approximately 1.3-fold of the *S. italica* Yugu1 genome.

**Fig. 3:**
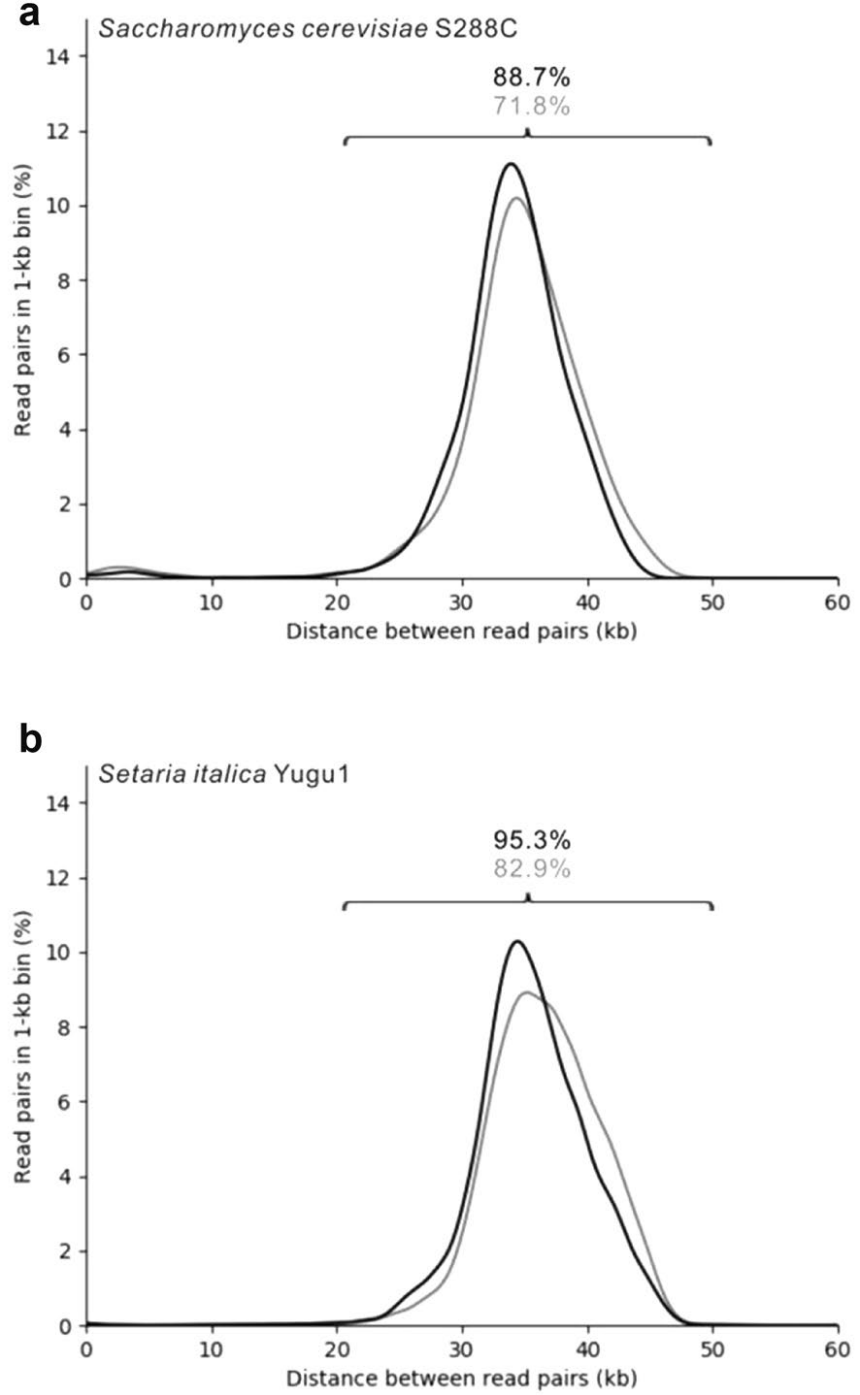
Length distribution of genomic distance spanned by Fosmid-size paired-end sequences. Smoothed histograms of the spacing between unique read pairs in Fosmid size paired-end libraries are shown for the *S. cerevisiae* S288C library Y1 (grey) and Y2 (black) (A) and the *S. italica* Yugu1 library S1 (grey) and S2 (black) (B) aganist their respective reference genomes. The y-axis represents percentage of all unique read pairs that fall in the 1-kb bin. The x-axis represents the distance between read pairs

Those paired ends located in unexpected spacing or orientation, e.g., spacing < 20 kb, > 50 kb, inverted orientation, tandem orientation and linking 2 reference contigs, were identified as chimaeras and counted (Additional file 2: Table S1). The chimaeric rate of unique read pairs (1157) in the nonredundant set of Y1 was 27.1% (Fig. 3a), and the chimaeric rate of unique read pairs (2663) in the nonredundant set of S1 was 16.6% (Fig. 3b).

### Further modification of the Fosmid vector based on pHZAUFOS2

In the pHZAUFOS2 -based method above, the two I*-*SceI sites were used to release the complete paired ends. However, the resulting complete pHZAUFOS2 vector band was ~ 8 kb (Additional file 1: Figure S4), which was just within the 5–10 kb range of the paired-end DNA fragments we recovered (Additional file 1: Figure S5A). This is why we had high vector contamination rates in the datasets of Y1 and S1. Therefore, to reduce the vector contamination rate and increase the effective paired-end data, we introduced another two I*-*SceI sites into the skeleton of pHZAUFOS2 without affecting its function. This was accomplished with two pairs of PCR primers P5 and P6 and P7 and P8 (Additional file 1: Figure S1). The new version of the vector was named pHZAUFOS3 (Fig. 2a). Then, we constructed the libraries Y2 (10× physical genome coverage) and S2 (20× physical genome coverage) in the pHZAUFOS3 vector. Digestion of the pHZAUFOS3 libraries with I*-*SceI resulted in complete inserts and 2–3 kb of vector pieces (Additional file 1: Figure S5B).

### Optimization of the method for Fosmid long paired-end sequencing

Our preliminary test data showed that too many chimaeras were introduced during Fosmid and/or paired-end library constructions. For large-insert library construction, the trapped small DNA fragments in the size-selected large fragment fractions used for library construction were usually the main cause of chimaeras. The higher the DNA fragment concentration loaded on the PFGE gel, the more the small DNA fragments were trapped.

To reduce chimaeras as much as possible, we took several measures for the construction of another two Fosmid libraries/paired-end libraries series: Y2 for *S. cerevisiae* S288C and S2 for *S. italica* Yugu1. First, we screened DNA fragments twice on PFGE gels in both the Fosmid library and paired-end library constructions to reduce the trapping of small fragments. In contrast to the paired-end library constructions of Y1 and S1, we dephosphorylated the paired-end fragments and ligated them to the phosphorylated Amp tag to reduce the ligation of the unrelated small DNA fragments. As a result, the chimaeric rates of Y2 and S2 were reduced to 10.6% and 4.2% compared to 27.1% and 16.6% of Y1 and S1, respectively (Fig. 3). The numbers of nonredundant 20- to 50-kb jumps from Y2 and S2 were 1518 (88.7%) and 9363 (95.3%), respectively. Moreover, we sought to generate more effective paired-end data at lower sequencing costs by increasing the physical coverage of the Fosmid library clones in each pool. Therefore, a total of ~ 0.2 million clones of S2 (20× physical genome coverage) were used to construct a paired-end library and sequenced in one PacBio flow cell, which generated 9363 unique Fosmid-size jumps, approximately the same as the number of S1 generated from six PacBio flow cells (Additional file 2: Table S1). A detailed breakdown of the sequencing reads from all four test libraries is available in Additional file 2: Table S1.

### Impact on de novo genome assemblies of whole genome PacBio reads

We tested the effect of Fosmid long paired-end sequences with long-range connectivities on de novo genome assemblies of whole-genome PacBio reads. First, we tested the effect of simulated Fosmid long paired-end data on de novo genome assemblies of simulated whole-genome PacBio subreads. We simulated the sequencing data of the *S. cerevisiae* S288C strain on the PacBio Sequal platform based on the reference genome of the *S. cerevisiae* S288C strain from NCBI (GCF_000263155.2) at different sequencing depths, 10×, 20×, 30×, 40× and 50×, and assembled five draft yeast genomes, Pb10, Pb20, Pb30, Pb40 and Pb50, respectively (Additional file 2: Table S2). Additionally, we simulated five yeast Fosmid libraries with insert sizes of 38 kb and a standard deviation of 2.2 kb at different genome physical coverages (10×, 20×, 30×, 40×, 50×) and correspondingly simulated five Fosimd long paired-end sequence sets (Fos10, Fos20, Fos30, Fos40, Fos50) generated by PacBio, with read lengths of 7 kb (paired ends) and a standard deviation of 2 kb (Additional file 2: Table S3). We reassembled Pb10, Pb20, Pb30, Pb40 and Pb50 by adding the simulated paired-end data of Fos10, Fos20, Fos30, Fos40 and Fos50, respectively. The results showed that the assembly quality improved as the sequencing depth of the genome increased and the physical genome coverage of the Fosmid library increased. Notably, when the sequencing depth of the genome reached 20× and the physical genome coverage of the Fosmid library reached 10× , the assembly quality significantly improved. All chromosomes were assembled completely and covered by one scaffold except chromosome 12 (Additional file 1: Figure S6A). Moreover, the assembly result reached chromosome level when the sequencing depth reached 30× and the physical genome coverage of the Fosmid library reached 20× (Additional file 1: Figure S6B).

**Fig. 4:**
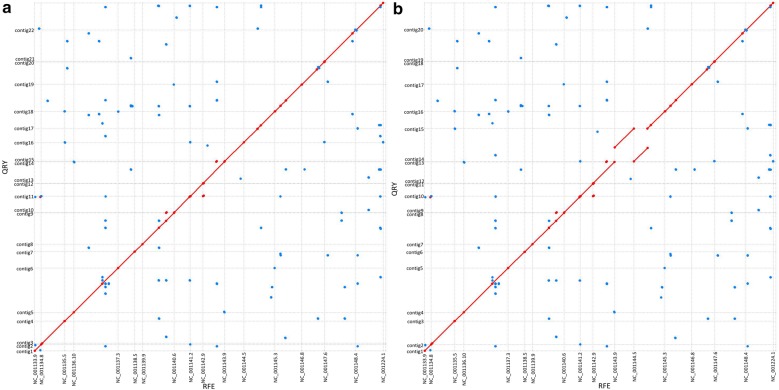
Genome alignments between scaffolds and reference. A is the comparison results of the assembly from the simulated sequencing depth of 30 × and the Y1 Fosmid long paired ends covering tenfold of the *S. cerevisiae* S288C physical genome with reference. B is the comparison results of the assembly from the simulated sequencing depth of 30 × and the Y2 Fosmid long paired-ends covering fivefold of the *S. cerevisiae* S288C physical genome with reference. The plot shows the best (1-to-1) alignments between the reference (x-axis) and each assembly (y-axis). Red lines indicate forward-strand matches while blue lines indicate reverse-complement matches. Dashed vertical lines delineate chromosome ends while dashed horizontal lines delineate contigs. A diagonal indicates concordant matches while off-diagonal matches indicate assembly errors or differences versus the reference

Then, we tested the effect of our real Fosmid long paired-end data on the de novo yeast genome assembly with the simulated whole-genome PacBio subreads. Of the five draft yeast genomes that were de novo assembled only by simulated PacBio whole-genome sequencing data, Pb30 had the average assembly quality. However, it only had an N50 scaffold length of 568 kb. When we added our real long paired-end data, Y1 (tenfold physical subread coverage) and Y2 (fivefold physical subread coverage), to improve the qualities of the draft yeast genome Pb30 (details see additional file 1: Table S4), the N50 of the assembled scaffold improved to 935 kb (Fig. 4a) and 786 kb (Fig. 4b), respectively.

Finally, we tested the effect of our real *S. italica* Yugu1 Fosmid long paired-end data on the de novo assembly of real whole genome PacBio subreads of the *S. italica* genome. Although the *S. italica* Yugu1 genome was published as the foxtail millet reference genome, it was assembled with Sanger reads. Therefore, we made use of the PacBio contigs of *S. italica* Yugu18, which has a genome sequence that is almost the same as that of *S. italica* Yugu1 (GWHABGJ00000000). The contig number of Yugu18 was 383, and the N50 length was 3.75 Mb. After adding our long paired-end sequences of S1 and S2 together (~ tenfold Fosmid physical subread coverage and ~ 1.5-fold whole-genome subread coverage), the scaffold number of *S. italica* Yugu18 was reduced to 330, and the N50 length was increased to 5.2 Mb, which was a 1.5-fold improvement to the assembly of the WGS only (Table 2).

**Table 2:** Summarized statistics for the assembly of *Setaria italica* Yugu18

Name	num_seqs	sum_len	avg_len	max_len	N50	< 30 kb
Yugu18_contigs	383	407,498,629	1,063,965	12,402,311	3,758,082	165
Yugu18_scaffold	330	407,887,709	1,236,023	14,943,871	5,196,440	164

Yugu18_ contigs: assembly of the whole-genome sequences from PacBio only

Yugu18_scaffold: assembly of the whole-genome sequences from PacBio and the long paired ends of S1 and S2

### Detection of structural rearrangements or assembly errors

One important application of jumping libraries is the comprehensive detection of chromosomal structural rearrangements/variants or assembly errors. Large fragment sizes enable the identification of uniquely aligned reads in both ends, particularly when the chromosomal structural variants or assembly errors are likely mediated by repetitive elements [30]. We used the S2 data of *S. italica* Yugu1 to detect the structural variants or assembly errors of the published *S. italica* Yugu1 genome sequence, which is used as the foxtail millet reference genome sequence. After filtering out the low-quality sequences, almost 50,000 unambiguous Fosmid-size subread paired ends were obtained that covered the genome sequence of approximately 0.75×. These paired ends with a left-end length of 2.85 kb and a right-end length of 2.85 kb on average were mapped to the Yugu1 genome sequence. Five distinct large rearrangements or assembly errors of dozens of kb were identified with 9 or more independent supporting subread pairs for each. These rearrangements or assembly errors included the three most frequently observed events—deletion, duplication and translocation (Table 3). A large deletion (~ 58 kb) was detected in chromosome VIII and framed by 12 unique subread pairs. This deletion was located at the end of the chromosome and without any annotation.

Our approach generated long paired ends and single ends. The length reached up to 2–3 kb for each end of paired-ends and 5 kb for single ends on average. Therefore, we took advantage of the long ends to detect small structural rearrangements or assembly errors. Five different rearrangements or assembly errors of several kb, including inversion, deletion and duplication, were detected with 7 or more unique supporting reads for each (Table 4).

**Table 3:** Examples of rearrangements in the Yugu1 genomeidentified by long paired ends

Support read number (non-duplicate)	Support read number (duplicate)	SV type	SV length (bp)	Coordinate (bp)
12	81	Deletion	58,435	Chr: NC_028457.1 39834375–39892810
11	32	Duplication	33,248	Chr: NC_028455.1 29621517–29654765
10	47	Duplication	49,573	Chr: NC_028452.1 16482798–16532371
10	43	Translocation	Non	NC_028452.1 28454982 NC_028451.1 596035
9	59	Translocation	Non	NC_028453.1 23754472 NC_028452.1 6538935

SV: structural arrangement; Coordinate: the location of SVs; NC_: chromosome; NW: scaffold

**Table 4:** Examples of rearrangements in the Yugu1 genome identified by long single ends

Support read number (non-duplicate)	Support read number (duplicate)	SV type	SV length (bp)	Coordinate (bp)
15	45	Inversion	8091	Chr: NC_028458.1 49998519–50006610
12	17	Deletion	1596	Chr: NC_028451.1 33653189–33654785
9	47	Duplication	1883	NW_014576740.1 62365–64247
7	37	Deletion	359	Chr: NC_028450.1 25710225–25710584
7	11	Duplication	2400	Chr: NC_028455.1 4360170–4362570

SV: structural arrangement; Coordinate: the location of SVs; NC_: chromosome; NW: scaffold

Large-insert paired ends also play an important role in comparative genomics studies [24, 48]. We aligned the long paired-ends of S2 to the *S. italica* Yugu1 and *S. italica* Yugu18 genome assemblies. The rate of nonredundant jumps located in the range of 20–50 kb was 96.0% in Yugu1 and 93.0% in Yugu18 genome (Additional file 2: Table S5).

## Discussion

We developed a new method for long paired-end sequencing of large DNA fragment libraries that could be complimentary to other methods, such as Fosill, pBACode, 10× Genomics, Hi-C and BioNano, to improve de novo genome assembly and detect structural arrangements and assembly errors.

Paired-ends from large DNA fragment libraries, such as Fosmid and BAC library, are usually used for detecting structural rearrangements/variants and assembly errors, delineating associated breakpoints and assisting de novo genome assembly. Their large spans help to resolve long repeats and segmental duplications and provide long-range connectivity to shotgun assemblies of complex genomes [22, 49, 50]. Several high-throughput paired-end sequencing approaches using large-insert genomic libraries, such as the Fosmid library called Fosill (Fosmid libraries by Illumina) [51] and the BAC library called pBACode [52], were developed and used for the de novo assembly and SV detection of several genomes [52, 53]. Also, large insert size paired-ends methods that do not depend on large-insert genomic libraries have been created for large and complex genomes, especially those rich in repeats, such as 10× Genomics [38], Hi-C [54], and BioNano [11, 46]. They make a significant contribution to the assembly of complex genomes [4, 55, 56], closing gaps [57, 58] and detecting structural variations [59] or large scale errors, such as those in pseudomolecules spanning chromosomes [60], including insertions, deletions, duplications and inversions spanning tens to hundreds of kb. However, these strategies based on massively parallel genome-sequencing technologies can not produce end sequences much longer than 1 kb. Therefore, the paired-ends generated by these methods are usually too short and require much higher physical coverages for partial compensation. Single-molecule real-time synthesis and sequencing technologies such as PacBio [6, 7] and Nanopore [8–10] are leading to a new era of biological sequencing. It is suitable for assisting de novo genome assembly via overlap-consensus methods, especially for large and complex genomes. Recently, the single-molecule real-time synthesis and sequencing technology is significantly improved and the error rate of it can be reduced to the level as NGS [61]. Our method applied the characteristics of large inserts of genomic libraries and long subreads of the PacBio platform to generate DNA calipers with long spans of 20–50 kb and long paired ends of up to 2–3 kb each end on average. These paired ends are much longer than those generated by other methods, and would become longer as the average subread length increases in the single-molecule real-time synthesis and sequencing technology. Since these long paired ends better tolerate sequencing errors, the positioning of sequences can be more precise, and the connection error of contigs can be reduced. Besides, the long-distance ends can be used to correct assembly errors of complex genomes [33–35]. The longer DNA read lengths can significantly increase the detection rate of structural rearrangement events and reduce the rate of mismatching with low physical coverage, especially for genomes containing high-repeat regions [62]. Moreover, our method results in a certain proportion of single ends; these long single ends (average > 5 kb) can be used as whole-genome sequences to detect small structural variants of tens to thousands of bp. In the application of our long Fosmid-size paired-end method with only ~ tenfold Fosmid physical subread coverage and ~ 1.5-fold whole-genome sequence subread coverage, five distinct large rearrangements or assembly errors of dozens of kb were identified with 9 or more independent supporting subread pairs for each (Table 2), and five small different rearrangements or assembly errors of several kb were detected with 7 or more unique supporting long single subread ends for each (Table 3). All of these large and small rearrangements of *S. italica* Yugu1 may imply misassemblies in the Yugu1 reference genome.

It has been shown that the rate of concordant jumps in which two ends were aligned to the same scaffold with correct spacing and orientation is an important parameter for the quality of paired-end methods. This parameter was 95.3% in our optimized method (Fig. 3). It is almost the same as the previously reported 96% of Fosill [51] and better than the 90.2% of pBACode [52]. Chimaeras were the main factor affecting this parameter and are usually an obstacle in the application of paired-end technology, which could result in misassemblies. In our study, we performed DNA fragment size selection twice on pulse field gel both in constructing the Fosmid library and paired-end library and ligated dephosphorylated paired-end fragments to phosphorylated Amp tag. By this measure, the chimaera rate of S2 significantly decreased to 4.2% (Fig. 3; Additional file 2: Table S2). The chimaeric rates of Y1 and Y2 were higher than those of S1 and S2 (Fig. 3). This phenomenon is most likely because the DNA concentration of the *S. cerevisiae* S288C loaded on the pulsed field electrophoresis gel was too high (much higher than that of *S. italica* Yugu1) to separate well (not shown) and can be avoided in future practice.

The conventional 40 kb mate-pair library was constructed by enzyme digestion [63]. The uneven distribution of the restriction sites might produce cloning bias. In Fosill method, Fosmid-size paired-end library was constructed with nick translation that could reduce the cloning bias [51]. However, this method depends on the delicate concentration of DNA polymerase I and dNTPs and has a limit in generating long paired ends. In pBACode method, a random-barcode-based high-throughput approach with ultrasonic interruption was used for BAC paired-end sequencing [52]. This approach can generate single ends of up to 800 bp long and pair them with the same barcode. All above three methods are based on Illumina technology that generate short end reads and are incompatible with emerging long-read high-throughput technologies [64, 65]. They usually use biotin labels [66] for recovering paired ends and/or use enzyme sites [67] to screen the positive paired ends. Paired-end sequencing samples are prepared by inverse PCR. However, the rate of base errors introduced by PCR will increase as amplification and insert size increase. This is incompatible with long-read technologies (> 10 kb). We instead adopt mechanical randomly interrupted DNA to effectively reduce bias and obtain uniform long ends. Our method is straightforward and easy to manipulate. In our study, paired-end samples were prepared through cloning and vector removal instead of PCR, and additional base errors and bias can be avoided. The *ampicillin* resistance gene was used both as a marker to screen positive long paired-end clones together with the vector *chloramphenicol* resistant gene (CmR) and as a tag to distinguish the left and right ends. The latter is highly important in paired-end sequencing methods, especially those generating long reads based on PacBio or Nanopore sequencing technologies. In addition, there are many options for tags used in this method, such as different antibiotic genes or one antibiotic gene with a random sequence of several bp as indexes. The indexes are very important in pooling samples of different libraries. Moreover, if random-barcode pairs such as pBACode [52] are introduced into our vector, pHZAUFOS3, our method can also distinguish different clones in pools to construct high-quality physical maps.

To adapt long-read single molecule sequencing technologies and generate long paired ends, we modified the vector based on pcc2FOS. Previously available Fosmid vectors usually use *Not*I digestion for insert sizing and release. For large DNA-insert clones from high GC content organisms or monocotyledonous plant genomes, digestion with *Not*I would cut each insert into several to many fragments, which makes insert sizing difficult and the release of intact inserts almost impossible [21]. In our new vector, pHZAUFOS3, four I*-*SceI sites were introduced, and the *chloramphenicol* resistant gene (CmR) and replicon (*ori*V) necessary for colony growth were located near to the two sides of the cloning site. Since I*-*SceI is a rare-cut restriction enzyme that recognizes an 18-bp sequence, the recognition sequence was not found in most species when searching the genome sequence database. Two I-SceI sites that flank the cloning site in the vector can be used to release complete large DNA inserts. Another two I*-*SceI sites located in the skeleton of the vector can fragment the vectors into pieces with lengths that are much shorter than those of the paired ends, ans so can effectively reduce the vector contamination rate and increase the effective paired-end data (Additional file 1: Figure S5). Adjusting the positions of the CmR and *ori*V can help to increase the proportion of the paired-end fragments to single-end fragments in the paired-end libraries.

It is well known that single molecule sequencing technologies such as PacBio and Oxford Nanopore Technologies can produce long read length sequences with an average length of more than 10 kb, but have a reduced accuracy (75–90%) due to their dependence on single-molecules detection [50, 68]. As the high error rate, the long-read technologies are rarely used to detect SNVs or indels. In these technologies, CCS derives a consensus sequence from noisy individual subreads [69, 70]. Recently, a long high-fidelity (HiFi) technology has been used to produce highly accurate (99.8%) HiFi reads of 13.5 kb in average length and applied for variant detection [61]. However, this technology is limited by the number of passes required to achieve the desired accuracy and the polymerase read length of the sequencing platform. Thus, the insert of the CCS library can’t be too long. The paired-ends generated by our method were shorter than 15 kb, which is in the range of the insert of the CCS library for HiFi sequencing. If our method is applied to the HiFi technology, it might generate highly accurate (99%) fosmid paired-ends that could be used to provide validation to structural variant calls. Moreover, in order to obtain longer connective information, we are attempting to apply our method to BAC paired-ends production. In fact, our vector pHZAUFOS3 can be used to construct both Fosmid and BAC libraries (our unpublished data). We believe that the highly accurate long BAC paired-ends could be used to further improve the quality of genome assembly and make the detection of large-scale structural variations more accurate and efficient.

## Conclusion

We developed a new method for obtaining long spanning long paired ends. This method is straightforward and enables DNA manipulation to be performed easily. It can be applied complimentary to other methods in assembling complex genomes, detecting structural variations and assembly errors, and assessing assembly qualities.

## Methods

### Construction and preparation of the pHZAUFOS2 and pHZAUFOS3 vectors based on pcc2FOS

PCR primers (P1: 5-attaccctgttatccctaGTCGGGGCTGGCTTAACTAT-3′ and P2: 5-attaccctgttatccctaTTCGCGTTGGCCGATTCATT-3′) containing the I*-*SceI sites were used to amplify the *Lac*Z fragment based on the pcc2FOS vector. The resulting fragment was named the A fragment. The pcc2FOS vector was *Not*I digested, and then the pcc2FOS skeleton without *Lac*Z was recovered, self-ligated and propagated in *E. coli* EPI300.-T1R (Epicentre). The new PCR primers (P3: 5′-ATTCAAATCGTTTTCGTTACCGC-3′ and P4: 5′-ATGCCTTCAGGAACAATAGAAATCT-3′) complementary to the area between *ori*V and CmR were used to generate the new skeleton of the vector pcc2FOS, named B. These two PCR products, A and B, were ligated and then transformed into *E. coli* strain EPI300.-T1R (Epicentre), resulting pHZAUFOS2. Transformants were cultured on LB plates with 12.5 μg/mL chloramphenicol, 80 μg/mL X-gal and 100 μg/mL IPTG overnight before counting and collecting.

Two more I*-*SceI sites were introduced into pHZAUFOS2 by PCR with primers (P5: 5-GGTTGTATGCCTGCTGTGGA-3′ and P6: 5-CGCTCAGCGCAAGAAGAAAT-3′ and P7: 5-tagggataacagggtaatGCGCTGAGCGTAAGAGCTA-3′ and P8: 5-tagggataacagggtaatCACACCGAGGTTACTCCGTT-3′). The PCR products were ligated and transformed into E. coli strain EPI300.-T1R (Epicentre), resulting pHZAUFOS3. Transformants were cultured on LB plates with 12.5 μg/mL chloramphenicol, 80 μg/mL X-gal and 100 μg/mL IPTG overnight before counting and collecting.

pHZAUFOS2 and pHZAUFOS3 plasmid DNA were propagated in *E. coli* strain EPI300.-T1R (Epicentre) grown at 37 °C in LB broth with shaking (225–250 rpm), 12.5 μg/mL chloramphenicol and the 500× Copy Control Fosmid Autoinduction Solution overnight (16–20 h). Plasmid DNA was prepared using the plasmid midi kit (Qiagen) according to the manufacturer’s instructions. Vectors were prepared as described by Shi et al. [21] for BAC library construction. Plasmid DNA (40 µg) was linearized using 200 units *Eco72*I restriction endonucleases from Fermentas at 37 °C for 2 h, dephosphorylated by a two-step incubation (1 h at 37 °C and 1 h at 55 °C) with 2 × 25 units calf intestine alkaline phosphatase (NEB), self-ligated at 16 °C overnight, separated on a CHEF agarose gel. The linear vector fragments were recovered by electroelution. The undigested circular plasmid DNA and/or re-ligated non-dephosphorylated vector DNA will be removed in this process. Ultra-0.5 centrifugal filter devices (Amicon) were used to concentrate the linear vectors up to a final concentration of 0.5 mg/μL.

### Fosmid library construction

Fosmid libraries were constructed using the method modified from the protocol of Copy Control™ HTP Fosmid Library Production Kit with pCC2FOS™ Vector (Epicentre). High molecular weight genomic DNA was prepared as described by Shi et al. [21] for BAC library construction. Liquid culture and young leaves were used for yeast *S. cerevisia*e strain S288C and *S. italica* Yugu1, respectively. Yeast protoplasts and *S. italica* Yugu1 nuclei were evenly embedded in the gel plugs of low melting temperature agarose. The gel plugs were then treated with proteinase K for 48 h at 50 °C and partially sheared by freezing and thawing (20 s freeze and 45 s thaw). The DNA fragments were size-selected twice by pulsed-field gel electrophoresis. The DNA fragments of 30–45 kb were recovered, end repaired, ligated to the vector and then packaged with the MaxPlax Packaging Extract [20]. The packaged products were used to infect EPI300-T1R cells (Epicentre) and then the transfected cells were spread on LB plates with 12.5 μg/mL chloramphenicol, 80 μg/mL X-gal and 100 μg/mL IPTG. After incubation at 37 °C overnight, the clones were washed off plates using liquid LB, pooled together and then stored at ‒ 80 °C.

### Fosmid paired-end sequencing library construction

Pooled Fosmid clones were cultured and induced to a high copy number in the 500× Copy Control Fosmid Autoinduction Solution (Epicentre) at 37 °C overnight (16–20 h) with 12.5 μg/mL chloramphenicol and shaking (225–250 rpm). Then DNA was extracted by alkaline lysis method and purified by phenol: chloroform: isoamyl alcohol (25:24:1).

A total of 400 μg of pooled Fosmid DNA was sheared into fragments by g-TUBE (Covaris), with a mean size ranging from 6 to 20 kb. All DNA samples were loaded into a united single well in the middle of the gel and the markers on the wells of the two sides, and separated twice on CHEF apparatus at 0.5–1.5 s linear ramp, 9 V/cm, 14 °C in 0.5× TBE buffer for 15–17 h. The gel fraction of 12–18 kb was recovered from the unstained center part of the gel. DNA fragments were electroeluted at 4 °C in 1× TAE buffer, concentrated by Ultra-0.5 centrifugal filter devices (Amicon) and dephosphorylated by a two-step incubation (1 h at 37 °C and 1 h at 55 °C) with 2 × 25 units calf intestine alkaline phosphatase (NEB). Phenol:chloroform:isoamyl alcohol (25:24:1) was used to remove the calf intestine alkaline phosphatase (NEB). The supernatant was concentrated and purified by Ultra-0.5 centrifugal filter devices (Amicon). A total of 200 μL DNA was end repaired with 50 units of T4 DNA polymerase (ThermoFisher), 100 units of Klenow Fragment (ThermoFisher) in 500 μL reaction mixture [10× Klenow Fragment buffer, 200 μM of dNTP Mix] and incubated at 37 °C for 1 h. The reaction mix was incubated at 65 °C for 15 min to terminate the end repairing and treated with phenol:chloroform:isoamyl alcohol (25:24:1). The supernatant was concentrated and purified by Ultra-0.5 centrifugal filter devices (Amicon) again*.*

The Amp resistance gene fragment was amplified by PCR from the plasmid puc19 with the phosphorylated primers (5-AAACGCGCGAGACGAAAGGG-3′ and 5-GGGGTCTGACGCTCAGTGGA-3′). The PCR products were purified through glue recycling and concentrated by Amicon® Ultra-0.5 centrifugal filter devices to a final concentration of 0.5 mg/µL. The end repaired DNA fragments (30 μL) were ligated with the Amp resistance gene fragments (1:10) with 10 units T4 DNA ligase (ThermoFisher) in a final volume of 50 μL at 16 °C for 16–18 h. After incubated at 65 °C for 15 min to terminate the reaction, the ligation mix was used to transform TransforMax™ EPI300™ Electrocompetent *E. coli* (Epicentre) by electroporation. The tranformants were spread on LB plates with 12.5 μg/mL chloramphenicol, 50 μg/mL carbenicillin, 80 μg/mL X-gal and 100 μg/mL IPTG. After incubation at 37 °C overnight, the clones were washed off plates using liquid LB, pooled together and then stored at ‒ 80 °C.

### Preparation of Fosmid long paired-ends for sequencing

The pooled clones of Fosmid paired-end sequencing library were cultured and induced to a high copy number by the 500× Copy Control Fosmid Autoinduction Solution (Epicentre) at 37 °C overnight (16–20 h) with 12.5 μg/mL chloramphenicol and 50 μg/mL carbenicillin and shaking (225–250 rpm). Plasmid DNA was extracted using the plasmid large constructed kit (Qiagen) according to the manufacturer’s instructions, digested with I*-*SceI, and separated on agarose gel. The paired-end fragment fractions of 5–10 kb were recovered, electroeluted and purified. The final samples were concentrated by Ultra-0.5 centrifugal filter devices (Amicon) to a final amount of 30 μg and sent to Frasergen Company for sequencing on the PacBio Sequel platform.

### Fosmid paired-end sequence analysis

PacBio subreads were corrected by SMRT Link Software (v5.1.0) ccs (v3.0.0) (ccs --polish --richQVs --numThreads 16 --minPasses 2). Fosmid end sequences should contain a part of the vector sequence in both ends, so they were extracted by BLASTn (v2.7.1+ ) [71] based on the following features: (1) VES1(Vector end sequence 1) was 348 bp; (2) VES2 (Vector end sequence 2) was 300 bp; and (3) the *Ampicillin* resistance gene tag was 1218 bp. The paired reads of FESs (Fosmid end sequences) were aligned to the *S. cerevisiae* strain S288C (GCF_000146045.2) or *S. italica* Yugu1 (GCF_000263155.2) or *S. italic* Yugu18 (GWHABGJ00000000) genome sequences by bwa (v0.7.17) [72]. The single reads of FESs were aligned independently with bwaaln (-k17 -W40 -r10 -A1 -O1 -E1 -L0). MergeBam Alignments, from the picard package (https://picard.sourceforge.net/) v1.59, were used to return the unmapped reads to the aligned BAM file. A custom picard module was used to classify the reads based on the definitions described by Williams et al. [51]: (1) unambiguously mapped read pairs: pairs with both reads aligned with a mapping quality score > 0 as assigned by BWA; (2) duplicate read pairs: pairs where both reads have identical start sites of forward and reverse sequencing reads; (3) correct jumps: read pairs where the reads face each other and are aligned 20–50 kb apart; (4) chimaeric jumps: (a) pairs with unexpected orientation (inverted read pairs facing away from each other and tandem reads aligning to the same strand in the same orientation); and (b) pairs with unexpected spacing (> 100 kb or aligning to different contigs in the reference genome sequence, usually different chromosomes).

### De novo genome assembly

The sequencing data of the *S. cerevisiae* S288C strain and *S. italica* Yugu1 on the PacBio Sequal platform with sequencing depths of 10× , 20× , 30× , 40× , 50× were simulated by NPBSS software (v1.0.3) (--accuracy- Mean 0.90 --length-mean 15,000 --model_qcmodel_qc_clr) [73]. Canu (v1.7) [74] was used for the de novo assembly of the data. BLASTn (v2.7.1+ ) was used to adjust the order and direction of the assembled contigs and map the contigs to the *S. cerevisiae* strain S288C (GCF_000146045.2) or *S. italica* Yugu1 (GCF_000263155.2) reference genome sequence. The highest alignment result of each contig was extracted and sorted according to the positive and negative chain alignment and the coordinate starting position. DNAdiff (v1.3) [75] was used to verify and evaluate the assembled contigs against the reference genome. NUCmer (v3.1) (-l 100 -c 1000) [76] was used to compare the sorted contigs with the reference genome sequence. The mummerplot (v3.5) was used to draw the dotplot map (—large —png). SeqKit (v0.10.0) (stats -a) [77] was used to measure the contig assembly results.

The Fosmid long paired-end sequences were aligned to the simulated contigs by minimap2 (v2.11) (-a -x map-pb) [78]; low-quality reads were removed, and chimaeras alignment results were generated by samtools (v1.3) (view -h -q 60 -F 2048) [79]. Paired-end sequences with a mass alignment value of 60 without chimaericas were retained. The software bamToBed (v2.27.0) [80] was used to obtain the alignment coordinate information, and the longest paired-end was retained after calculating the total length of the multiple paired-end sequences from one clone. Then, the retained paired end sequences were combined with the simulated contigs to assemble the scaffolds by SSPACE (v3.0) (-k 2 -p 1) [81]. The order and direction of the assembled scaffolds were adjusted, and the scaffolds were aligned to the *S. cerevisiae* strain S288C (GCF_000146045.2) or *S. italica* Yugu1 (GCF_000263155.2) reference genome sequences to assess the assembly quality.

### SV detection

After the low-quality data was filtered out by samtools (v1.8), the long paired ends were aligned to the reference genome sequences by bwa, and then, the data were transferred from bam file to deduplication by sambamba (0.6.7). Large structual arrangements were detected by Delly (0.8.1). Small SVs were detected by sniffles (v1.0.10) using the long single ends including those split from the paired ends as PacBio whole-genome sequencing subreads.

## Supplementary information

**Additional file 1: Figure S1.** The modification process of pHZAUFOS2 and pHZAUFOS3.** Figure S2.** The putative clone types of the pcc2FOS paired-end library. **Figure S3.** The map of the vector pHZAUFOS2. **Figure S4.** Sequence read length distribution of preprocessed PacBio sequencing data. **Figure S5.** I-SceI digestion of the random clones from the pHZAUFOS2 (A) and pHZAUFOS3 (B) paired-end libraries DNA. **Figure S6.** Simulated yeast genome alignments between the scaffolds and reference.

**Additional file 2: Table S1.** Detailed breakdown of sequencing reads. **Table S2.** Statistics of yeast contig assembly by simulation. **Table S3.** Statistics of simulated yeast Fosmid libraries and paired-end reads. **Table S4.** Statistics of scaffold assembly by simulated PacBio data and our Y1 and Y2 long paired ends. **Table S5.** Assessment of genome assemblies.

## Data Availability

The datasets used and/or analyzed during the current study are available from the corresponding author on reasonable request. The *S. cerevisiae* strain S288C (GCF_000146045.2) and *S. italica* Yugu1 (GCF_000263155.2) reference genome sequences can be found in NCBI data base. The PacBio data of the *S. italica* Yugu18 can be found in the Genome Warehouse in BIG Data Center with the accession number GWHABGJ00000000. The Fosmid paired-end raw data of the *S. cerevisiae* S288C and *S. italica* Yugu1 are deposited in NCBI’S Sequence Read Archive (SRA) with accession code PRJNA580081.

## Abbreviations

SVs: structural rearrangements or variants
Cm: chloramphenicol
CmR: chloramphenicol resistance gene
Amp: ampicillin
BAC: bacterial artificial chromosome
PFGE: pulse field gel electrophoresis
WGS: whole genome sequencing
NGS: next-generation sequencing
IPTG: isopropyl-β-d-thiogalactoside
X-gal: 5-bromo-4-chloro-3-indolyl-β-d-galactopyranoside
VES: vector end sequence
FES: fosmid end sequence
