# Supplementary material for: High-throughput long paired-end sequencing of a Fosmid library by PacBio
Source: Plant Methods. 2019 Nov 26;15:142. doi: 10.1186/s13007-019-0525-6 (PMC6878638; doi:10.1186/s13007-019-0525-6)
Supplement: Supplementary file 1 — Additional file 1: Figure S1. The modification process of pHZAUFOS2 and pHZAUFOS3. Figure S2. The putative clone types of the pcc2FOS paired-end library. Figure S3. The map of the vector pHZAUFOS2. Figure S4. Sequence read length distribution of preprocessed PacBio sequencing data. Figure S5. I-SceI digestion of the random clones from the pHZAUFOS2 (A) and pHZAUFOS3 (B) paired-end libraries DNA. Figure S6. Simulated yeast genome alignments between the scaffolds and reference. [file 13007_2019_525_MOESM1_ESM.docx]

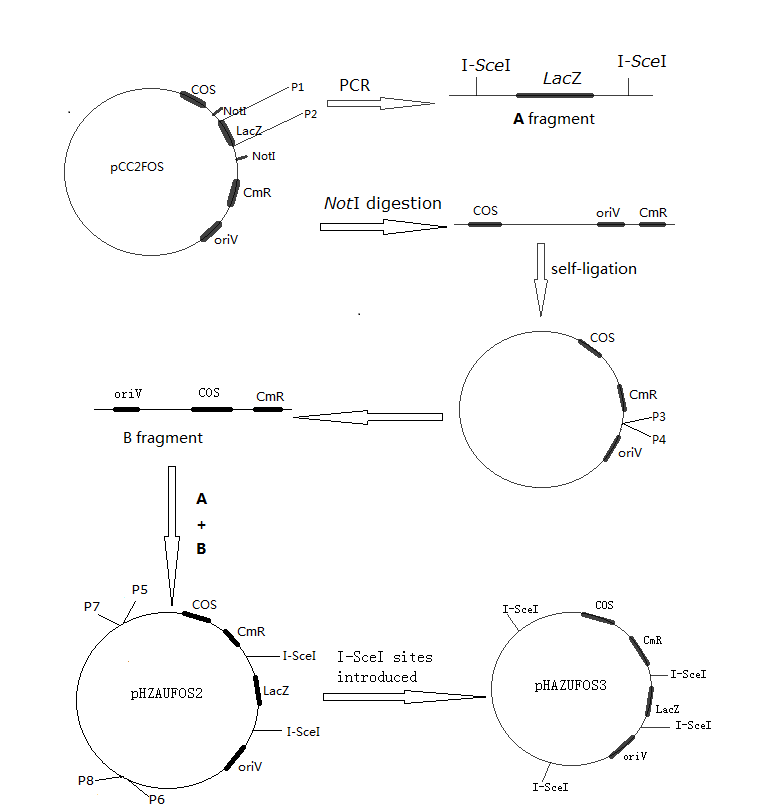


Figure S1 The modification process of pHZAUFOS2 and pHZAUFOS3

PCR primers (P1 and P2) containing the I*-Sce*I sites were used to amplify the *Lac*Z fragment based on the pcc2FOS vector. The resulting fragment was named the A fragment. The pcc2FOS vector was *Not*I digested, and then the pcc2FOS skeleton without LacZ was recovered, self-ligated and propagated. The new PCR primers (P3 and P4) complementary to the area between *ori*V and CmR were used to generate the new skeleton of the vector pcc2FOS, named B. These two PCR products, A and B, were ligated, resulting pHZAUFOS2. Two more I-*Sce*I sites were introduced into pHZAUFOS2 by PCR with primers (P5 and P6 and P7 and P8). The PCR products were ligated, resulting pHZAUFOS3.


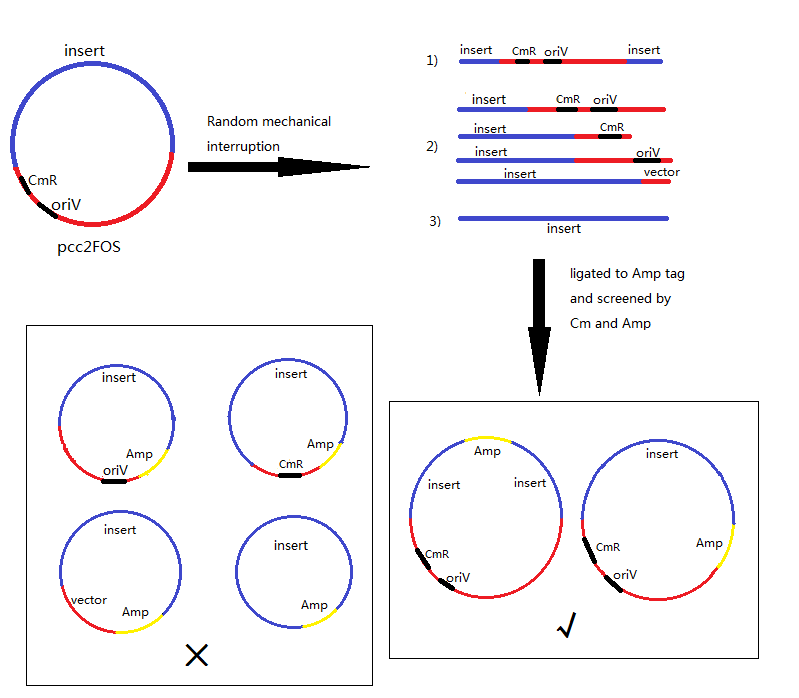


Figure S2 The putative clone types of the pcc2FOS paired-end library

Fosmid DNA was broken into several fragments by random mechanical interruption: 1) Fragments containing the entire vector sequence and the paired-end insert sequence, 2) fragments containing part of or the entire vector sequence and single-end insert sequence, and 3) fragments containing only the insert sequence. After ligated to *Ampicillin* resistance gene tags and screened by Cm and Amp double antibiotics, only positive paired-end and single-end clones containing the *ori*V and CmR in vectors could be screened out (√). The others were eliminated (×).


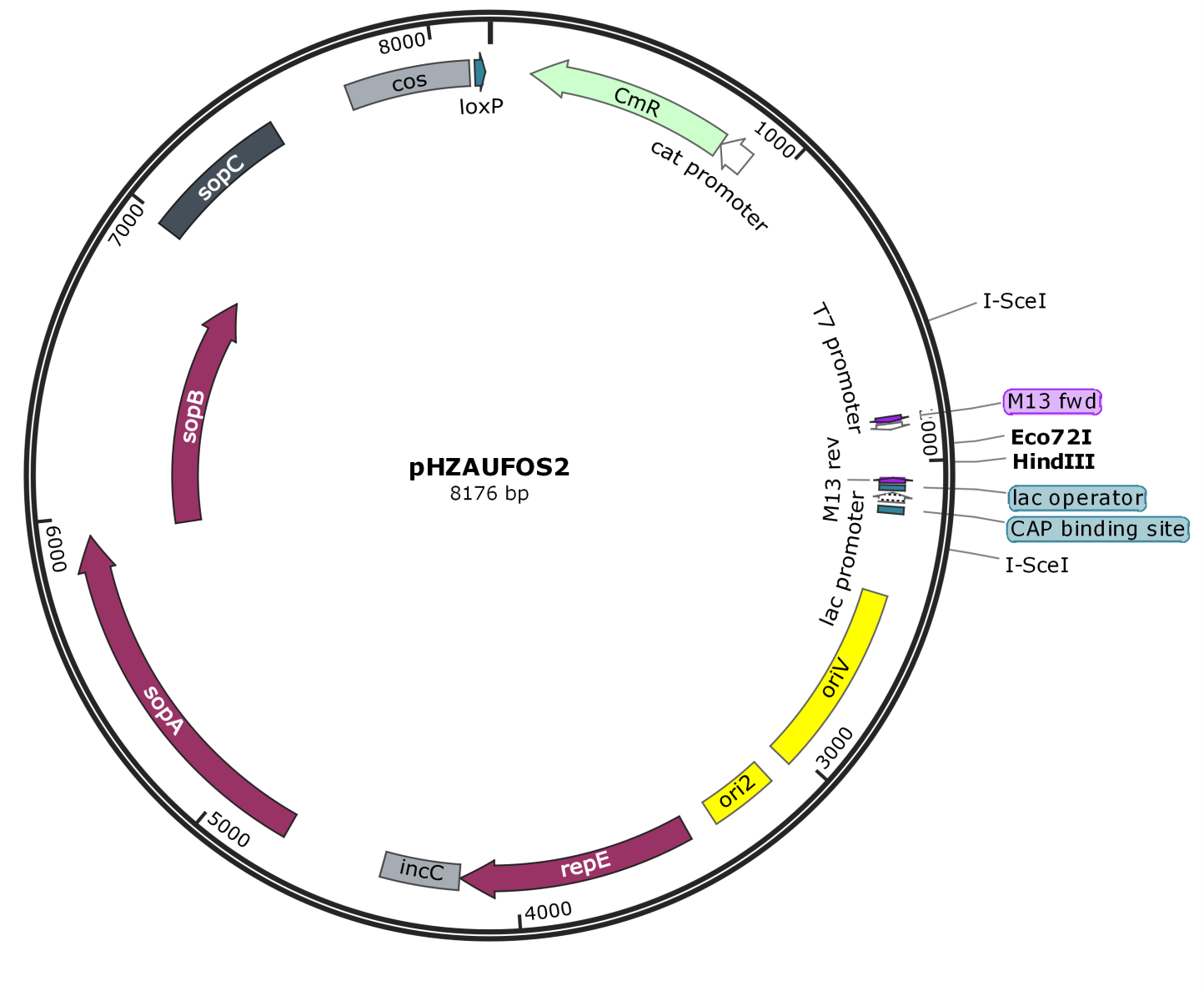


Figure S3 The map of the vector pHZAUFOS2

The *Lac*Z fragment was moved between CmR and *ori*V, and two I*-Sce*I sites flanking the *Lac*Z fragment were used to test the insert size.

*
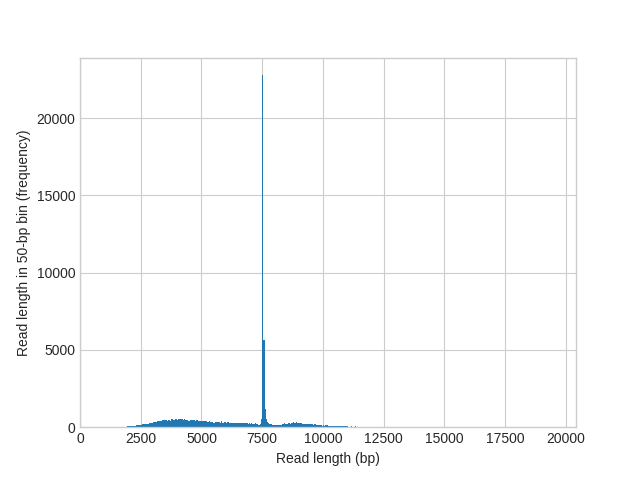
*

Figure S4 Sequence read length distribution of preprocessed PacBio sequencing data

The paired-end read lengths of PacBio were 2-10 kb. The peak in the read length at 7.5 kb was corresponds to the length of pHZAUFOS2vector backbone.


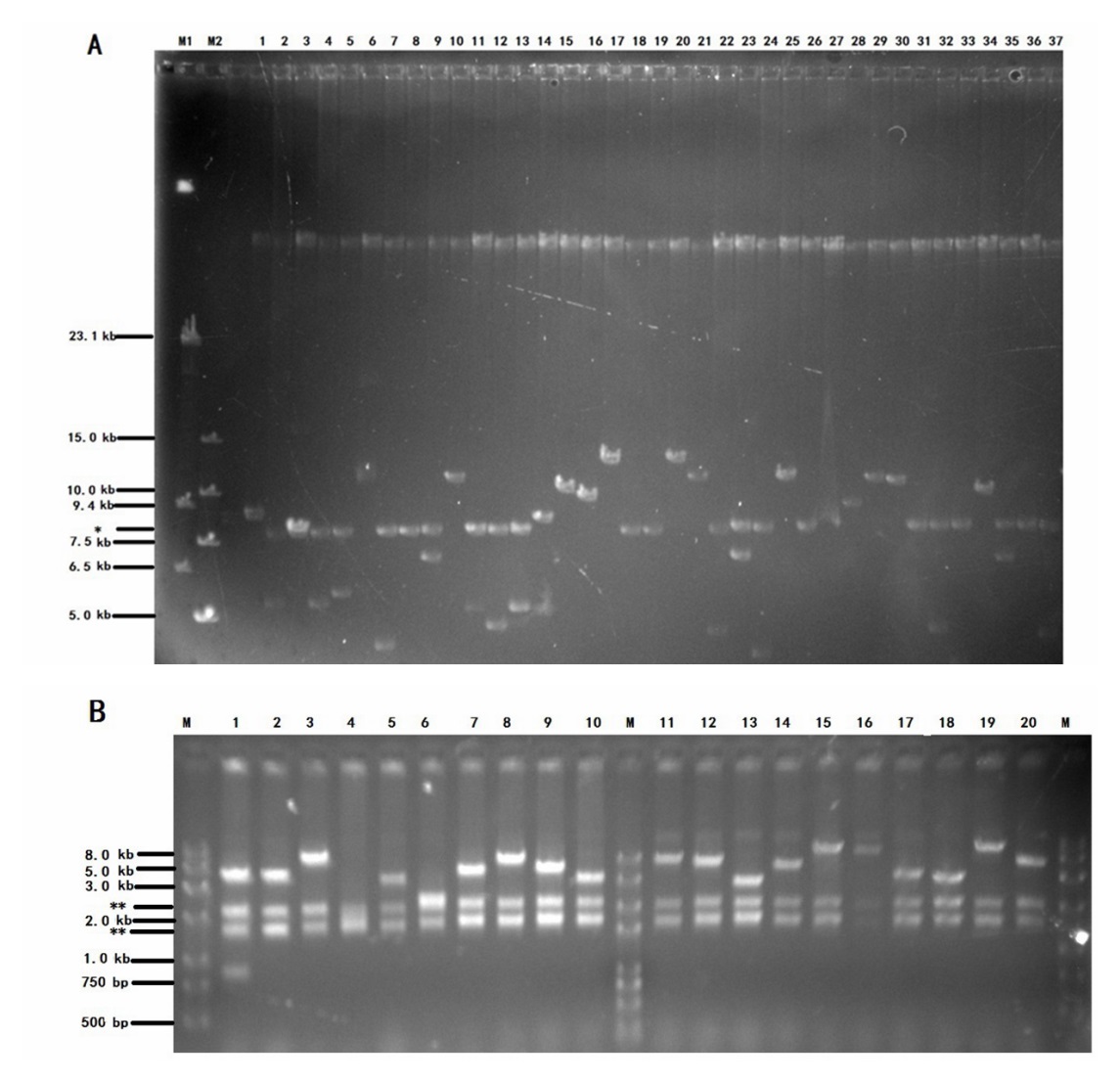


Figure S5 I*-Sce*I digestion of the random clones from the pHZAUFOS2 (A) and pHZAUFOS3 (B) paired-end libraries DNA

M1 is the lambda DNA *Hind*III digestion bands, and M2 is the *Trans*15K DNA ladder. * represents the pHZAUFOS2 vector band of 7,524 bp, which is just within the 5-10 kb range of the paired-end DNA fragments recovered for sequencing. M is the *Tans*2K^@^ Plus II DNA ladder. ** represents the fragmented pHZAUFOS3 vector bands.


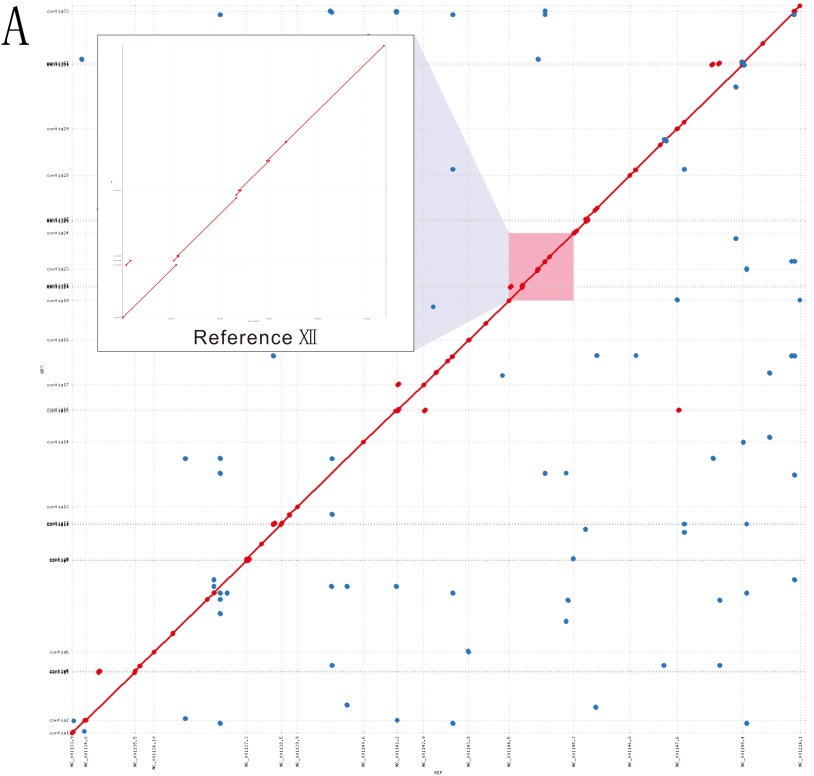

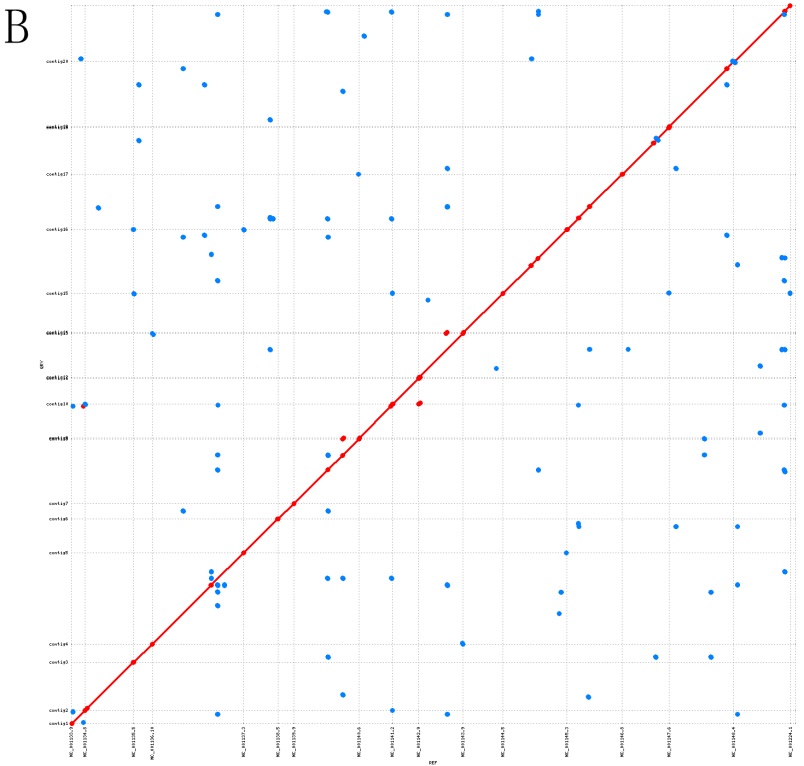


Figure S6 Simulated yeast genome alignments between the scaffolds and reference

A is the comparison results of the assembly from the simulated PacBio data at the depth of 20x and the simulated Fosmid long paired ends covering 10-fold of the *S. cerevisiae* S288C physical genome with reference. The small pictures show the result of chromosome 12 alignment. B is the comparison results of the assembly from the simulated PacBio data at the depth of 30x and the simulated Fosmid long paired-ends covering 20-fold of the *S. cerevisiae* S288C physical genome with reference. The plot shows the best (1-to-1) alignments between the reference (x-axis) and each assembly (y-axis). Red lines indicate forward-strand matches while blue lines indicate reverse-complement matches. Dashed vertical lines delineate chromosome ends while dashed horizontal lines delineate contigs. A diagonal indicates concordant matches while off-diagonal matches indicate assembly errors or differences versus the reference.
