# Supplementary material for: High-throughput long paired-end sequencing of a Fosmid library by PacBio
Source: Plant Methods. 2019 Nov 26;15:142. doi: 10.1186/s13007-019-0525-6 (PMC6878638; doi:10.1186/s13007-019-0525-6)
Supplement: Supplementary file 2 — Additional file 2: Table S1. Detailed breakdown of sequencing reads. Table S2. Statistics of yeast contig assembly by simulation. Table S3. Statistics of simulated yeast Fosmid libraries and paired-end reads. Table S4. Statistics of scaffold assembly by simulated PacBio data and our Y1 and Y2 long paired ends. Table S5. Assessment of genome assemblies. [file 13007_2019_525_MOESM2_ESM.docx]

Table S1 Detailed breakdown of sequencing reads

| Organism: | *Saccharomyces cerevisiae* S288C (Y1) | | *Saccharomyces cerevisiae* S288C (Y2) | | *Setaria italica* Yugu1 (S1) | | *Setaria italica* Yugu1 (S2) | |  |
| --- | --- | --- | --- | --- | --- | --- | --- | --- | --- |
| **Total read pairs** | **35,510** | **100.0%** | **17,844** | **100.0%** | **67,220** | **100.0%** | **75,047** | **100.0%** | |
| Unaligned pairs | 340 | 1.0% | 67 | 0.4% | 111 | 0.2% | 1,238 | 1.6% | |
| Only one read aligned | 980 | 2.8% | 388 | 2.2% | 962 | 1.4% | 4,946 | 6.6% | |
| Chimaeric alignment | 2,986 | 8.4% | 218 | 1.2% | 10,295 | 15.3% | 2,118 | 2.8% | |
| Multiple placements | 8,048 | 22.7% | 3,359 | 18.8% | 22,622 | 33.7% | 44,846 | 59.8% | |
| Unambiguously placed pairs^a^ | 25,812 | 72.7% | 14,644 | 82.1% | 41,988 | 62.5% | 52,030 | 69.3% | |
| **Unambiguously placed pairs^a^** | **25,812** | **100.0%** | **14,644** | **100.0%** | **41,988** | **100.0%** | **52,030** | **100.0%** | |
| Chimaeric pairs | 3,485 | 13.5% | 759 | 5.2% | 4,817 | 11.4% | 1,702 | 3.3% | |
| inverted orientation | 108 |  | 15 |  | 266 |  | 101 |  | |
| tandem orientation | 145 |  | 26 |  | 210 |  | 246 |  | |
| linking 2 reference contigs | 3,207 |  | 704 |  | 4,198 |  | 1,324 |  | |
| spacing >100 kb | 25 |  | 14 |  | 143 |  | 31 |  | |
| Spaced <20 kb or 50-100 kb | 135 | 0.5% | 64 | 0.4% | 202 | 0.5% | 472 | 0.9% | |
| Pairs with correct spacing and orientation^b^ | 22,192 | 86.0% | 13821 | 94.4% | 36,969 | 88.0% | 49,856 | 95.8% | |
| **Total unique pairs^c^** | **4,273** | **100.0%** | **1712** | **100.0%** | **16,085** | **100.0%** | **9,824** | **100.0%** | |
| Unique chimaeric pairs | 1,157 | 27.1% | 182 | 10.6% | 2,663 | 16.6% | 413 | 4.2% | |
| inverted orientation | 44 |  | 7 |  | 145 |  | 23 |  | |
| tandem orientation | 49 |  | 9 |  | 111 |  | 47 |  | |
| linking 2 reference contigs | 1,050 |  | 161 |  | 2,338 |  | 331 |  | |
| spacing >100 kb | 14 |  | 5 |  | 69 |  | 12 |  | |
| Unique pairs spaced 1-20 kb or 50-100 kb | 49 | 1.1% | 11 | 0.6% | 87 | 0.6% | 48 | 0.5% | |
| Unique pairs spaced <1 kb | 0 | 0.0% | 1 | 0.1% | 1 | 0.1% | 0 | 0.0% | |
| Unique pairs with correct spacing and orientation | 3,067 | 71.8% | 1,518 | 88.7% | 13,334 | 82.9% | 9,363 | 95.3% | |

^a^Read pairs with both reads aligned to a single region in the genome.

^b^Convergent read pairs that aligned 20–50 kb apart.

^c^After removal of duplicate read pairs (within each Fosmid size paired-end library) with identical start sites of forward and reverse sequencing reads.

Table S2 Statistics of yeast contig assembly by simulation

|  | Pb10 | Pb20 | Pb30 | Pb40 | Pb50 | Ref. |
| --- | --- | --- | --- | --- | --- | --- |
| N50 (bp) | 271,692 | 415,200 | 568,643 | 916,050 | 918,063 | 924,431 |
| NG50 (bp) | 271,692 | 415,200 | 568,643 | 916,050 | 918,063 | 924,431 |
| Total length (bp) | 11,873,804 | 12,224,892 | 12,112,945 | 12,045,788 | 12,078,349 | 12,157,105 |
| Total number | 75 | 54 | 32 | 20 | 21 | 17 |
| Max length (bp) | 734,864 | 1,087,633 | 992,654 | 1,529,661 | 1,526,200 | 1,531,933 |
| Number of length<30 kb | 13 | 15 | 4 | 4 | 5 | 0 |
| Covered (%) | 96.61 | 98.20 | 98.56 | 98.67 | 98.66 | 100.00 |
| Break | 39 | 24 | 21 | 18 | 17 | 0 |

Pb10, Pb20, Pb30, Pb40 and Pb50 represent the simulated PacBio sequencing assemblies with depths of 10x, 20x, 30x, 40x, and 50x, respectively. Breakpoints were calculated as the sum of relocations, translocations, and inversions; covered (%) indicates genome coverage.

Table S3 Statistics of simulated yeast Fosmid libraries and paired-end reads

|  | Fos10 | Fos20 | Fos30 | Fos40 | Fos50 |
| --- | --- | --- | --- | --- | --- |
| Number of clones | 3,199 | 6,398 | 9,598 | 12,797 | 15,996 |
| Number of reads | 14,730 | 29,462 | 44,198 | 58,930 | 73,662 |
| Sequencing depth (duplicate) | 8.48 | 16.96 | 25.45 | 33.93 | 42.41 |
| Sequencing depth (non-duplicate) | 1.84 | 3.68 | 5.53 | 7.37 | 9.21 |
| Covered (%) | 84.12 | 97.48 | 99.60 | 99.94 | 99.99 |

Fos10, Fos20, Fos30, Fos40 and Fos50 represent Fosmid libraries with physical genome coverage of 10x, 20x, 30x, 40x, 50x, respectively.

Table S4 Statistics of scaffold assembly by simulated PacBio data and our Y1 and Y2 long paired ends

| Name | read_ nums | Unamb | mv_dup | pe_same_scaff | insert_mean | insert_std | N50 | NG50 | total_base | Nbase | num_scaff | max_len | Covered | break | len_less30kb |
| --- | --- | --- | --- | --- | --- | --- | --- | --- | --- | --- | --- | --- | --- | --- | --- |
| pb10X-yeast1 | 35510 | 25786 | 4655 | 52.91 | 35359 | 3751 | 802154 | 802154 | 12062428 | 188624 | 24 | 1514007 | 96.61 | 132 | 6 |
| Pb20X-yeast1 | 35510 | 26310 | 4641 | 59.0 | 35427 | 3754 | 788437 | 788437 | 12240891 | 15999 | 31 | 1537966 | 98.20 | 68 | 13 |
| Pb30X-yeast1 | 35510 | 26746 | 4762 | 60.69 | 35439 | 3752 | 935415 | 935415 | 12128013 | 15068 | 20 | 1539034 | 98.56 | 44 | 4 |
| Pb40X-yeast1 | 35510 | 27097 | 4815 | 61.80 | 35442 | 3763 | 916050 | 916050 | 12045788 | 0 | 20 | 1529661 | 98.67 | 18 | 4 |
| Pb50X-yeast1 | 35510 | 26939 | 4787 | 61.9 | 35440 | 3765 | 918063 | 918063 | 12078349 | 0 | 21 | 1526200 | 98.66 | 17 | 5 |
|  |  |  |  |  |  |  |  |  |  |  |  |  |  |  |  |
| pb10X-yeast2 | 17844 | 13698 | 1661 | 73.21 | 34486 | 3446 | 567463 | 567463 | 12045865 | 172061 | 31 | 1523829 | 96.61 | 122 | 9 |
| Pb20X-yeast2 | 17844 | 13843 | 1663 | 78.23 | 34541 | 3425 | 789669 | 789669 | 12247697 | 22805 | 33 | 1537219 | 98.20 | 63 | 14 |
| Pb30X-yeast2 | 17844 | 14002 | 1684 | 83.02 | 34554 | 3433 | 785683 | 785683 | 12112955 | 10 | 22 | 1539034 | 98.56 | 39 | 4 |
| Pb40X-yeast2 | 17844 | 14129 | 1701 | 84.6 | 34556 | 3442 | 916050 | 916050 | 12045788 | 0 | 20 | 1529661 | 98.67 | 18 | 4 |
| Pb50X-yeast2 | 17844 | 14124 | 1694 | 84.83 | 34559 | 3434 | 918063 | 918063 | 12078349 | 0 | 21 | 1526200 | 98.66 | 17 | 5 |

Table S5 Assessment of genome assemblies

| Organism: | *Setaria italica* Yugu1 | | *Setaria italica* Yugu18 | |
| --- | --- | --- | --- | --- |
| **Total read pairs** | **75,047** | **100.0%** | **75,047** | **100.0%** |
| Unaligned pairs | 1,250 | 1.7% | 1,253 | 1.7% |
| Only one read aligned | 5,799 | 7.7% | 6,243 | 8.3% |
| Chimaeric alignment | 887 | 1.2% | 2,766 | 3.7% |
| Multiple placements | 11,126 | 14.8% | 14,895 | 19.8% |
| Unambiguously placed pairs^a^ | 55,645 | 74.1% | 52,029 | 69.3% |
| **Unambiguously placed pairs**^a^ | **55,645** | **100.0%** | **52,029** | **100.0%** |
| Chimaeric pairs | 1,966 | 3.5% | 1,905 | 3.7% |
| inverted orientation | 88 |  | 148 |  |
| tandem orientation | 292 |  | 214 |  |
| linking 2 reference contigs | 1,531 |  | 1,488 |  |
| spacing >100 kb | 55 |  | 55 |  |
| Spaced <20 kb or 50-100 kb | 491 | 0.9% | 939 | 1.8% |
| Pairs with correct spacing and orientation^b^ | 53,188 | 95.6% | 49,185 | 94.5% |
| **Total unique pairs^c^** | **10,129** | **100.0%** | **9,710** | **100.0%** |
| Unique chimaeric pairs | 417 | 4.1% | 502 | 5.2% |
| inverted orientation | 19 |  | 52 |  |
| tandem orientation | 52 |  | 43 |  |
| linking 2 reference contigs | 332 |  | 389 |  |
| spacing >100 kb | 14 |  | 18 |  |
| Unique pairs spaced 1-20 kb or 50-100 kb | 56 | 0.6% | 176 | 1.8% |
| Unique pairs spaced <1 kb | 0 | 0.0% | 1 | 0.0% |
| Unique pairs with correct spacing and orientation | 9,719 | 96.0% | 9,031 | 93.0% |

^a^Read pairs with both reads aligned to a single region in the genome.

^b^Convergent read pairs that aligned 20–50 kb apart.

^c^After removal of duplicate read pairs (within each Fosmid size paired-end library) with identical start sites of forward and reverse sequencing reads.
